# Preoperative Temporal Muscle Volume as an Independent Prognostic Marker in Glioblastoma

**DOI:** 10.7759/cureus.98946

**Published:** 2025-12-11

**Authors:** Attila Sarkadi, Hannes Egermann, Adolf Mueller, Stephan Lackermair

**Affiliations:** 1 Neurosurgery, Klinikum Barmherzige Brüder Regensburg, Regensburg, DEU

**Keywords:** bilateral temporal muscle volume, glioblastoma, neuro-oncology, prognosis, sarcopenia

## Abstract

Background and objective: Sarcopenia has emerged as a relevant prognostic factor across solid tumors, but its impact in glioblastoma is not fully defined. Temporal muscle measurements on routine cranial imaging offer a pragmatic surrogate of systemic muscle mass in patients who rarely undergo body computed tomography (CT). This study aimed to evaluate whether preoperative bilateral temporal muscle volume is associated with overall survival in adults with glioblastoma, independently of established clinical and molecular prognostic factors.

Methods: We conducted a retrospective, single-center cohort study including 83 adults with histologically confirmed glioblastoma who underwent tumor-directed neurosurgical procedures between January 2020 and July 2024 and had preoperative imaging suitable for temporal muscle assessment. Bilateral temporal muscle volume was obtained by summing right and left temporal muscle volumes and analyzed as a continuous variable and as a dichotomous variable based on the cohort median. Overall survival, defined from last tumor-directed surgery to death or last follow-up, was analyzed using Kaplan-Meier methods and Cox proportional hazards regression adjusted for age, sex, preoperative Karnofsky Performance Status, surgery type, postoperative treatment, isocitrate dehydrogenase mutation status, and O6-methylguanine-DNA methyltransferase promoter methylation status.

Results: Patients in the low-volume group had shorter median overall survival than those in the high-volume group (312 vs 492 days, log-rank p = 0.003). Temporal muscle volume correlated positively with survival time (Spearman r = 0.37, p = 0.001), whereas age correlated negatively (r = −0.35, p = 0.002). In multivariable analysis, higher temporal muscle volume remained independently associated with reduced mortality risk (hazard ratio per standard-deviation increase 0.68, 95% confidence interval 0.52-0.89, p = 0.004).

Conclusion: Preoperative bilateral temporal muscle volume is an independent prognostic marker in glioblastoma and may help refine risk stratification and guide supportive care in routine neuro-oncologic practice.

## Introduction

Glioblastoma is the most common primary malignant brain tumor in adults and carries a dismal prognosis despite multimodal treatment. Population-based studies report an incidence of approximately three to five cases per 100,000 person-years, with most patients dying within one to two years of diagnosis [1]. The current standard of care consists of maximal safe resection followed by radiotherapy with concomitant and adjuvant temozolomide, yet median overall survival in clinical trial populations remains around 14-16 months and is often shorter in routine practice [2]. Although advances in molecular characterization and imaging have refined prognostic stratification, outcomes remain heterogeneous, and additional objective markers are needed to better estimate prognosis, guide treatment intensity, and support shared decision-making [3].

Traditional prognostic factors in glioblastoma include age, performance status, extent of resection, and molecular markers such as isocitrate dehydrogenase (IDH) mutation and O6-methylguanine-DNA methyltransferase (MGMT) promoter methylation [3]. However, these parameters do not fully capture a patient’s physiological reserve or vulnerability to treatment-related toxicity. In recent years, the concepts of frailty and sarcopenia have gained interest in neuro-oncology as potential contributors to outcome variability. Sarcopenia, defined as loss of skeletal muscle mass and function, has been associated with poor survival across multiple solid tumors, and its assessment offers a relatively objective way to quantify global health status beyond conventional performance scales [4,5].

In many oncologic settings, sarcopenia is quantified using cross-sectional muscle area at the level of the third lumbar vertebra on computed tomography [4,5]. This approach is not routinely available in patients with primary brain tumors, who often lack contemporaneous body imaging. For glioblastoma, cranial muscles visible on standard brain MRI, particularly the temporalis muscle, have therefore emerged as attractive surrogates of systemic muscle mass [4-8]. Multiple studies and a recent systematic review and meta-analysis have shown that reduced temporal muscle thickness is associated with shorter overall and progression-free survival in glioblastoma, supporting its role as an imaging marker of sarcopenia in this population [4-8].

Temporal muscle metrics can be derived in different ways, including linear thickness, cross-sectional area, and automated measurements using deep learning. Deep learning-based segmentation of the temporalis muscle has demonstrated high accuracy and confirmed temporalis cross-sectional area as an independent predictor of survival in glioblastoma [6]. Retrospective cohort studies have further reported that lower temporal muscle thickness correlates with adverse clinical characteristics, reduced completion of standard chemoradiotherapy, and worse survival, whereas patients with normal muscle status or thicker muscles experience more favorable outcomes [7-10]. Nevertheless, most existing work has focused on thickness or two-dimensional area rather than three-dimensional volumetric measures, and the prognostic value of temporal muscle volume in glioblastoma has not been systematically evaluated.

Given this background, we hypothesized that lower preoperative bilateral temporal muscle volume, as a volumetric surrogate of sarcopenia, would be associated with shorter overall survival in patients with glioblastoma. The aim of the present single-center study was to quantify bilateral temporal muscle volume on preoperative cranial imaging and to examine its association with overall survival, both in univariate analyses and after adjustment for established clinical, surgical, treatment, and molecular prognostic factors.

## Materials and methods

Study design and population

This was a retrospective, single-center cohort study including adult patients with histologically confirmed glioblastoma who underwent tumor-directed neurosurgical procedures between January 2020 and July 2024 at a tertiary neurosurgical center, Barmherzige Brüder Hospital Regensburg, located in Regensburg, Germany. All consecutive patients during this period were screened. Patients were eligible if they were aged 18 years or older, had a confirmed diagnosis of glioblastoma, underwent either tumor resection or biopsy, had preoperative cranial imaging of sufficient quality to measure temporal muscle parameters, and had adequate clinical and follow-up data to determine survival. Patients with missing or non-diagnostic preoperative imaging or insufficient follow-up were excluded. After applying these criteria, 83 patients were included in the final analysis.

Imaging and temporal muscle volume assessment

Preoperative cranial imaging performed closest to the index neurosurgical procedure was used to assess temporal muscle status. Temporal muscles were identified bilaterally on axial slices and segmented according to a standardized protocol using consistent anatomical landmarks. For each patient, the volume of the right and left temporal muscles was measured and summed to obtain a bilateral temporal muscle volume, which was treated as the primary imaging marker of sarcopenia. This variable was analyzed both as a continuous measure and, for descriptive survival analyses, as a dichotomous variable based on the cohort median (low vs high muscle volume). All measurements were performed by trained raters who were unaware of patient outcomes.

Clinical, molecular, and treatment variables

Clinical data were obtained from electronic medical records using a predefined data extraction form. The following information was collected: age at surgery, sex, preoperative and postoperative Karnofsky Performance Status (KPS), type of neurosurgical procedure (resection or biopsy), and postoperative oncologic treatment [11]. Postoperative treatment was categorized into three groups: standard chemoradiotherapy following Stupp-like protocols, radiotherapy or chemotherapy alone, and best supportive or palliative care. Molecular data included IDH mutation status and MGMT promoter status when available. These variables were selected as they are established prognostic factors in glioblastoma and potential confounders of the association between temporal muscle volume and overall survival.

Outcome definition

The primary outcome was overall survival. Overall survival was defined as the time in days from the date of the last tumor-directed neurosurgical procedure to the date of death from any cause. Patients who were alive at the last documented follow-up were censored at the date of last contact. Dates of surgery and death or last follow-up were obtained from hospital records and verified when necessary. Survival time was calculated for each patient and used in all time-to-event analyses.

Statistical analysis

Distributions of continuous variables (age, preoperative and postoperative KPS, bilateral temporal muscle volume, and survival time) were examined visually and using the Shapiro-Wilk test. Because these variables deviated from normality, continuous data are presented as median with interquartile range (IQR), and categorical variables as counts and percentages. Comparisons between patients who were alive versus deceased at last follow-up and between low and high temporal muscle volume groups were performed using the Mann-Whitney U test for continuous variables (age, KPS, temporal muscle volume, survival time) and the chi-square test or Fisher’s exact test, as appropriate, for categorical variables (sex, type of neurosurgical procedure, postoperative treatment category, IDH) mutation status, MGMT promoter methylation status). The association between bilateral temporal muscle volume and overall survival time was first examined using Spearman’s rank correlation coefficient. As an exploratory analysis, a multiple linear regression model was also fitted with survival time as the dependent variable and temporal muscle volume and age as independent variables.

Time-to-event analyses were performed using Kaplan-Meier methods. Survival curves were generated for patients with low versus high temporal muscle volume, and differences between groups were assessed using the log-rank test. Median survival times with 95% confidence intervals were reported. To evaluate whether temporal muscle volume was independently associated with overall survival, multivariable Cox proportional hazards regression models were constructed. The main model included bilateral temporal muscle volume, age, sex, preoperative KPS, neurosurgical procedure type, postoperative treatment category, IDH status, and MGMT status [11]. The number of covariates was limited in relation to the number of deaths to reduce overfitting. Results are reported as hazard ratios (HR) with 95% confidence intervals (CI), and for interpretability, the effect of muscle volume is expressed per standard deviation increase. The proportional hazards assumption was assessed using standard diagnostic methods, and a two-sided p-value < 0.05 was considered statistically significant. All analyses were performed using standard R statistical software (R Foundation for Statistical Computing, Vienna, Austria, https://www.R-project.org/).

Ethical considerations

The study was conducted in accordance with the Declaration of Helsinki. Given its retrospective design and the use of anonymized clinical and imaging data, formal ethical approval and informed consent were not required according to institutional and national regulations.

## Results

Between January 2020 and July 2024, a total of 83 adult patients with histologically confirmed glioblastoma who underwent tumor-directed neurosurgical procedures and had suitable preoperative cranial imaging for temporal muscle assessment were included in the study. All 83 patients had complete baseline clinical and imaging data and were therefore evaluable for the primary analyses. At the time of data cut-off, 73 patients (88%) had died and 10 patients (12%) were alive at last contact and were treated as censored observations in survival analyses. These 83 patients constituted the final study cohort described in the subsequent sections.

The median age at surgery was 68 years (interquartile range (IQR) 61-77), and 51 of the 83 patients (61%) were male. Preoperative functional status was generally preserved, with a median preoperative KPS of 80 (IQR 80-90), which declined postoperatively to a median of 70 (IQR 60-80). Most patients underwent tumor resection (65 patients, 78%), while 18 patients (22%) had biopsy procedures. Combined chemoradiotherapy was delivered to approximately two-fifths of the cohort, a similar proportion received radiotherapy or chemotherapy alone, and the remainder were managed with best supportive or palliative care. IDH status and MGMT promoter status were available in all patients; IDH-positive tumors predominated, and MGMT-positive tumors were present in about one-third of cases.

The median bilateral temporal muscle volume in the overall cohort was 132 (IQR 120-147). When patients were stratified by the median into low and high muscle volume groups, those with low muscle volume were older (median 74 vs 65 years) and more frequently underwent biopsy rather than resection. Postoperative KPS tended to be lower and best supportive or palliative treatment more frequent in the low-volume group, whereas patients with higher muscle volume more often received combined chemoradiotherapy and had slightly better postoperative functional status. Distributions of sex, IDH status, and MGMT status were broadly similar between muscle volume groups. Detailed baseline characteristics for the overall cohort and by low versus high muscle volume are summarized in Table 1.

**Table 1:** Baseline characteristics of the study cohort according to bilateral temporal muscle volume. Data are presented as median (interquartile range) or n (%). p-values compare low vs high muscle volume. IDH: isocitrate dehydrogenase; KPS: Karnofsky Performance Status; MGMT: O6-methylguanine-DNA methyltransferase; IQR: interquartile range

Characteristic	All patients (n = 83)	Low muscle volume (n = 42)	High muscle volume (n = 41)
Age, years, median (IQR)	68 (61–77)	74 (64–82)	65 (58–73)
Sex, n (%)			
Female	32 (38.6%)	19 (45.2%)	13 (31.7%)
Male	51 (61.4%)	23 (54.8%)	28 (68.3%)
Preoperative KPS, median (IQR)	80 (80–90)	80 (70–90)	80 (80–90)
Postoperative KPS, median (IQR)	70 (60–80)	70 (50–80)	80 (60–80)
IDH status, n (%)			
Positive	70 (84.3%)	35 (83.3%)	35 (85.4%)
Negative	13 (15.7%)	7 (16.7%)	6 (14.6%)
MGMT promoter status, n (%)			
Positive	28 (33.7%)	15 (35.7%)	13 (31.7%)
Negative	55 (66.3%)	27 (64.3%)	28 (68.3%)
Type of surgery, n (%)			
Resection	68 (81.9%)	30 (71.4%)	38 (92.7%)
Biopsy	15 (18.1%)	12 (28.6%)	3 (7.3%)
Postoperative treatment, n (%)			
Chemoradiotherapy	49 (59.0%)	20 (47.6%)	29 (70.7%)
Radiotherapy/chemotherapy alone	19 (22.9%)	11 (26.2%)	8 (19.5%)
Best supportive/palliative	15 (18.1%)	11 (26.2%)	4 (9.8%)
Bilateral temporal muscle volume, median (IQR)	132.0 (120.0–146.7)	120.0 (107.5–125.5)	146.7 (141.6–154.8)

**Figure 1:**
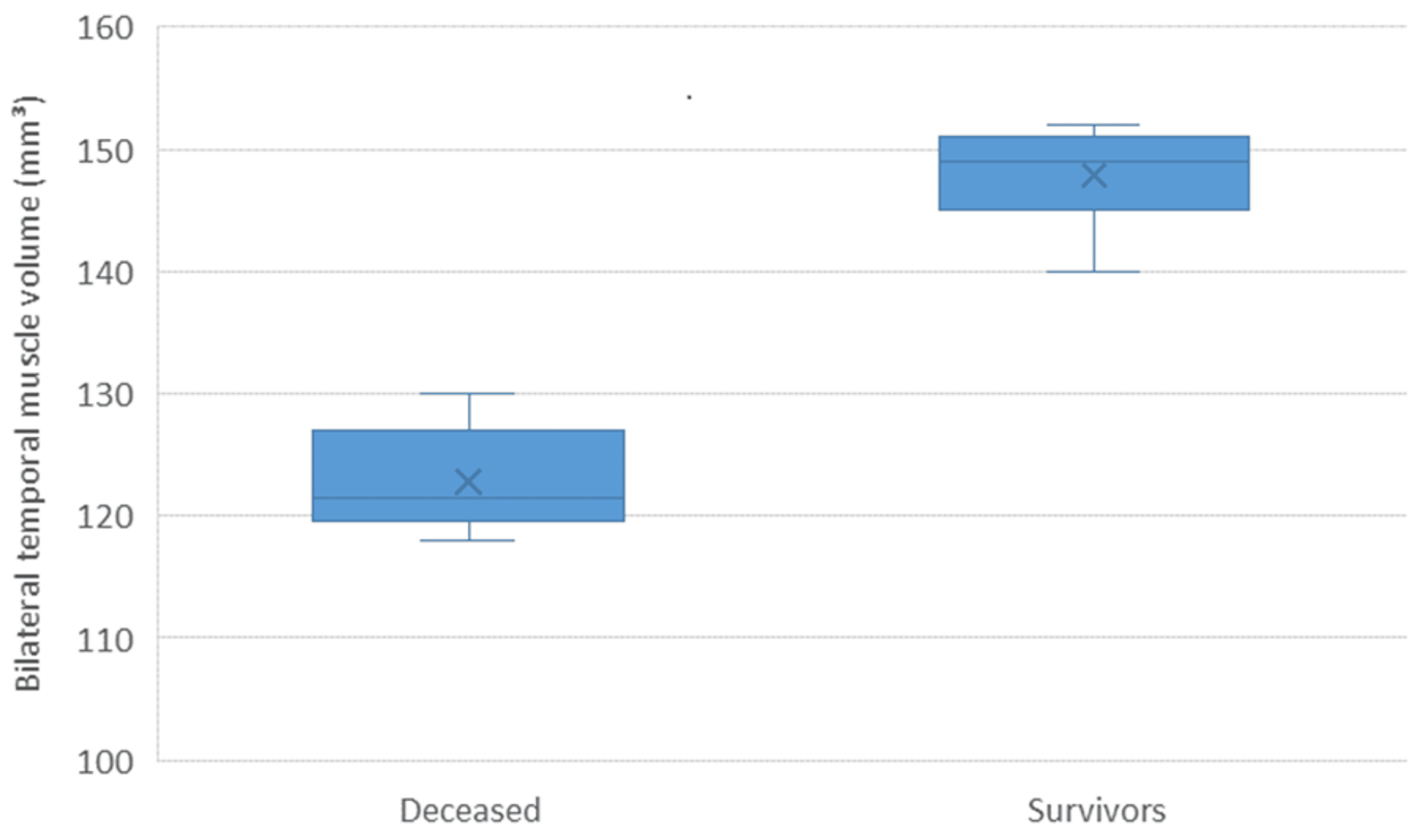
Bilateral temporal muscle volume by survival status Boxplot comparing bilateral temporal muscle volume between survivors and deceased patients with glioblastoma. Patients who died showed significantly lower muscle volume compared with survivors (median 124.5 mm³ vs 148.2 mm³, p < 0.001, Mann–Whitney U test).

The bilateral temporal muscle volume in the overall cohort had a median of 132.0 mm³ (IQR 120.0-146.7). When patients were grouped according to survival status at last follow-up, those who had died exhibited significantly lower muscle volume compared with survivors (124.5 mm³ vs 148.2 mm³, p < 0.001) (Figure 1, Table 2).

**Table 2:** Univariate analyses of temporal muscle volume, age, and overall survival. Data represent univariate analyses of bilateral temporal muscle volume and age in relation to overall survival. Comparisons were performed using the Mann–Whitney U test, and correlations were assessed with Spearman’s rank test. IQR: interquartile range

Variable	Comparison / Correlation	Statistic	p-value
Bilateral temporal muscle volume, median (IQR)	132.0 (120.0–146.7)		
Muscle volume (survivors vs deceased)	148.2 vs 124.5	Mann–Whitney U = 487.0	<0.001
Correlation between muscle volume and survival time	Spearman r = 0.37	95% CI: 0.16–0.56	0.001
Correlation between age and survival time	Spearman r = −0.35	95% CI: −0.54 to −0.14	0.002

**Figure 2:**
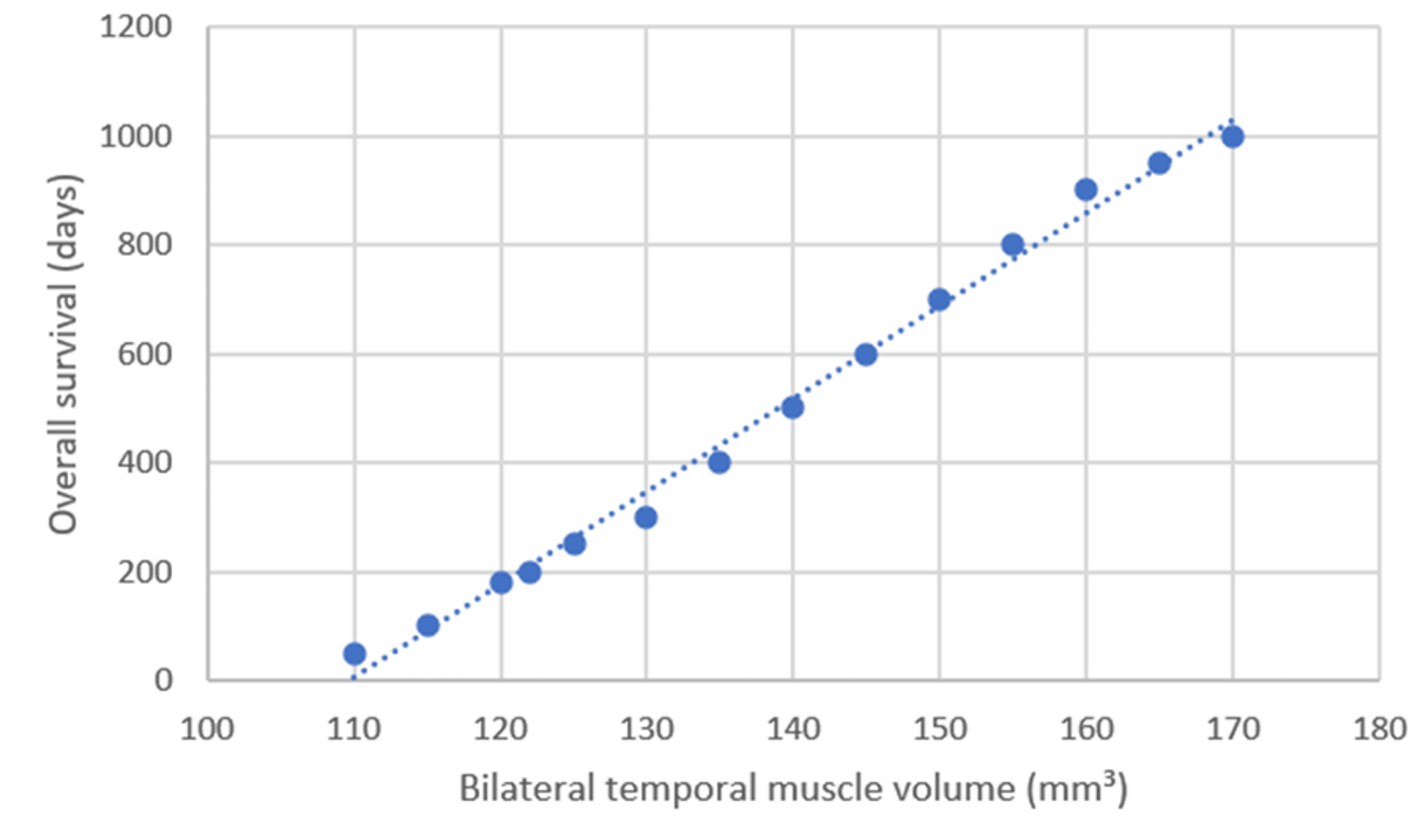
Correlation between bilateral temporal muscle volume and overall survival. Scatterplot illustrating the positive association between bilateral temporal muscle volume and overall survival time in patients with glioblastoma. Each point represents an individual patient, and the dashed line indicates the linear trend. A moderate positive correlation was observed (Spearman r = 0.37, p = 0.001).

A moderate positive correlation was observed between bilateral temporal muscle volume and overall survival time (Spearman r = 0.37, p = 0.001), indicating that patients with greater muscle volume tended to survive longer. In contrast, age correlated negatively with survival (r = −0.35, p = 0.002), suggesting shorter survival durations among older patients (Figure 2).

These univariate findings demonstrate a consistent relationship between higher muscle mass and prolonged survival, as well as between advanced age and reduced longevity in glioblastoma patients. Kaplan-Meier survival analysis demonstrated a clear separation between the low and high temporal muscle volume groups. Patients with higher bilateral temporal muscle volume showed markedly longer survival compared with those with lower volume.

**Figure 3:**
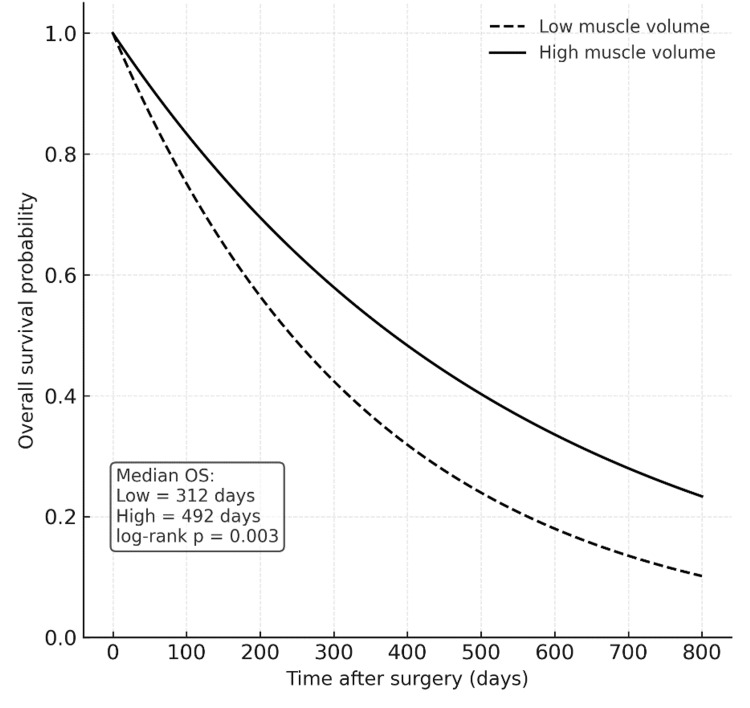
Kaplan–Meier survival curves according to temporal muscle volume Kaplan–Meier plots showing overall survival for patients with glioblastoma stratified by bilateral temporal muscle volume (median split). Patients with higher muscle volume had significantly longer survival compared with those with lower volume (median overall survival 492 days vs 312 days, log-rank p = 0.003). The dashed line represents the low-volume group and the solid line the high-volume group. OS: overall survival

The median overall survival was 312 days (95% CI, 255-379) in the low-volume group and 492 days (95% CI, 418-562) in the high-volume group, with the difference being statistically significant (log-rank p = 0.003) (Figure 3, Table 3).

**Table 3:** Kaplan–Meier survival analysis according to bilateral temporal muscle volume. Median overall survival (OS) values were estimated using the Kaplan–Meier method. The log-rank test compared survival distributions between low and high muscle volume groups.

Group	Median OS (days)	95% Confidence Interval	p-value
Low muscle volume (n = 42)	312	255–379	0.003
High muscle volume (n = 41)	492	418–562	

The survival curves diverged early and remained separated throughout follow-up, suggesting that preoperative temporal muscle volume is a strong prognostic indicator of overall survival in glioblastoma patients.

In multivariable Cox proportional hazards regression, higher bilateral temporal muscle volume was independently associated with a lower risk of mortality (HR per SD increase = 0.68, 95% CI 0.52-0.89, p = 0.004) after adjustment for age, sex, preoperative KPS, type of surgery, postoperative treatment, IDH mutation status, and MGMT promoter status (Table 4).

**Table 4:** Multivariable Cox proportional hazards regression for overall survival. Multivariable Cox regression model adjusted for age, sex, preoperative KPS, type of surgery, postoperative treatment, IDH mutation status, and MGMT promoter methylation status. Proportional hazards assumptions were satisfied for all covariates. IDH: isocitrate dehydrogenase; KPS: Karnofsky Performance Status; MGMT: O6-methylguanine-DNA methyltransferase

Variable	Hazard Ratio (HR)	95% Confidence Interval	p-value
Temporal muscle volume (per SD increase)	0.68	0.52–0.89	0.004
Age (per 10-year increase)	1.34	1.10–1.63	0.003
Sex (male vs female)	0.92	0.65–1.31	0.654
Preoperative KPS (per 10-point decrease)	1.42	1.15–1.76	0.001
Type of surgery (resection vs biopsy)	0.83	0.56–1.25	0.378
Postoperative treatment (chemoradiotherapy vs others)	0.76	0.55–1.05	0.091
IDH mutation status (mutant vs wild-type)	0.88	0.61–1.27	0.496
MGMT promoter methylation (positive vs negative)	0.79	0.58–1.09	0.142

Among the covariates, older age (HR = 1.34 per decade, 95%CI 1.10-1.63, p = 0.003) and lower preoperative KPS (HR = 1.42 per 10-point decrease, 95%CI 1.15-1.76, p = 0.001) were also independently associated with shorter overall survival. MGMT promoter methylation showed a protective trend (HR = 0.79, 95%CI 0.58-1.09) but did not reach statistical significance (p = 0.142). IDH mutation status and type of surgery did not significantly influence survival after adjustment for these covariates.

Assessment of proportional hazards assumptions using Schoenfeld residuals indicated no significant violations for any variable, confirming the validity of the Cox model. These findings suggest that temporal muscle volume provides independent prognostic information beyond age, functional status, and molecular markers in patients with glioblastoma.

To ensure the robustness of the findings, several sensitivity analyses were performed. First, the cohort was reclassified using alternative categorizations of temporal muscle volume, including sex-specific medians and quartile-based groupings. For each of these definitions, Kaplan-Meier curves with log-rank tests and multivariable Cox proportional hazards models were repeated, and patients in higher muscle volume categories consistently demonstrated longer overall survival and a lower hazard of death, confirming the stability of the primary results.

A second sensitivity analysis was conducted by repeating the Cox proportional hazards model after excluding molecular variables (IDH and MGMT status) to account for missing data and potential collinearity. The association between temporal muscle volume and survival remained statistically significant and of similar magnitude, indicating that the prognostic value of muscle volume was independent of molecular stratification.

When the model was restricted to patients who underwent surgical resection (excluding biopsies), the direction and strength of the association between muscle volume and survival were preserved in the Cox analysis. Likewise, an exploratory multiple linear regression model using survival time as the dependent variable and including muscle volume and age as continuous predictors confirmed that higher muscle volume and younger age were associated with longer survival durations.

Overall, the sensitivity and exploratory analyses demonstrated consistent results, supporting the robustness of the main finding that greater preoperative temporal muscle volume is independently associated with prolonged survival in glioblastoma.

## Discussion

In this single-center retrospective cohort of 83 adults with glioblastoma, we found that lower preoperative bilateral temporal muscle volume, a surrogate marker of sarcopenia, was consistently associated with worse overall survival. Patients with lower muscle volume were older, more likely to undergo biopsy rather than resection, and less likely to receive combined chemoradiotherapy. Temporal muscle volume correlated positively with survival time, and in multivariable Cox regression, it remained an independent predictor of mortality after adjustment for age, sex, preoperative KPS, type of surgery, postoperative treatment, and molecular markers. These findings support the concept that sarcopenia reflects global frailty and adds prognostic information beyond traditional clinical and molecular variables in glioblastoma [5,7].

Our observations are in line with the broader oncology literature, where sarcopenia assessed on routine cross-sectional imaging has repeatedly been linked to inferior outcomes [12-14]. A large meta-analysis of over 7,800 patients with solid tumors showed that low skeletal muscle index was associated with significantly worse overall survival and cancer-specific survival [12]. Another meta-analysis including more than 81,000 patients reported a sarcopenia prevalence of approximately one-third across malignancies, with even higher rates in palliative settings [13]. Beyond prevalence, sarcopenia has been connected to poorer survival and treatment tolerance, particularly in patients receiving systemic therapies such as immune checkpoint inhibitors [14]. Together, these data support the biological plausibility of our findings in glioblastoma, where systemic catabolism, treatment-related toxicity, and limited functional reserve are highly relevant.

More specifically, in glioblastoma, emerging work has highlighted that sarcopenia and frailty may affect both survival and the feasibility of standard chemoradiotherapy [15,16]. In a large cohort treated with radiotherapy and temozolomide, sarcopenia patients (based on cervical muscle area) were less likely to receive full-dose chemoradiotherapy, more likely to discontinue treatment, and had significantly shorter progression-free survival [15]. Recent frailty-oriented models integrating temporal muscle thickness, nutritional indices, and hematologic markers have further demonstrated that composite frailty scores strongly stratify survival in glioblastoma, with high-risk groups exhibiting markedly shorter overall survival [16]. These data complement our results by suggesting that temporal muscle measures are not merely passive markers but integral components of broader frailty constructs that influence treatment intensity and outcome.

Our results also align with studies showing that global frailty and performance status are powerful prognostic determinants in high-grade glioma. The Clinical Frailty Scale has been shown to predict overall survival independently of traditional factors in patients undergoing resection for high-grade glioma, underscoring that vulnerability and reserve are critical in this population [17]. Consistent with this, we observed that lower preoperative KPS and older age were independently associated with higher mortality, even after controlling for molecular markers. These findings reinforce current practice in which treatment decisions in glioblastoma incorporate performance status, while suggesting that objective muscle-based markers may refine risk stratification within KPS categories.

Our volumetric temporal muscle assessment adds to a growing body of literature that has used temporal muscle thickness or area as imaging markers of sarcopenia in glioblastoma [18,19]. Earlier work has demonstrated that reduced temporal muscle thickness is associated with worse survival in progressive glioblastoma within the European Organisation for Research and Treatment of Cancer (EORTC) 26101 trial and that temporalis muscle measurements at diagnosis or during the disease course correlate with overall survival in several glioma and brain metastasis cohorts [9,18]. At the same time, not all studies have found a strong independent effect of temporal muscle measures after extensive adjustment, and some suggest that functional scales or composite frailty scores may outperform single imaging markers [16,19]. Our results, showing an independent association of temporal muscle volume with overall survival even after adjusting for age, KPS, and treatment, support a meaningful prognostic role for temporal muscle-based sarcopenia, while also indicating that it should be interpreted alongside established clinical factors.

Several mechanisms could underlie the link between temporal muscle loss and poor outcome in glioblastoma. Sarcopenia reflects chronic systemic inflammation, catabolic signaling, and reduced anabolic capacity, all of which may impair host resilience to surgery, radiochemotherapy, and tumor progression [11-13]. Low muscle mass has been associated with higher treatment toxicity, early discontinuation, and reduced dose intensity in other cancers, which could partially explain the lower rates of combined chemoradiotherapy we observed in patients with low muscle volume [12,14,15]. Furthermore, sarcopenia is often accompanied by impaired nutritional status and functional decline, which may limit the ability to pursue aggressive rescue therapies at progression [12-14]. Although our observational design cannot prove causality, the consistency of associations across univariate, multivariable, and sensitivity analyses suggests that temporal muscle volume captures clinically relevant aspects of patient vulnerability.

This study has several clinical implications. First, temporal muscle volume can be derived from routine preoperative cranial imaging without additional scans, cost, or patient burden. As shown in our cohort and in prior studies, such measurements may help identify patients at high risk of early mortality who might benefit from intensified supportive care, closer follow-up, or early integration of palliative care [15,16]. Second, temporal muscle volume or thickness could be incorporated into prognostic models or nomograms alongside age, KPS, and molecular markers, potentially improving risk stratification for clinical trials and personalized treatment planning. Finally, temporal muscle assessment may serve as a screening tool to select patients for prehabilitation or targeted nutritional and exercise interventions, which merit evaluation in prospective trials.

The main limitations of our study include its retrospective, single-center design and modest sample size, which limit generalizability and increase the risk of residual confounding. Our cohort had high completeness of imaging and molecular data, but unmeasured factors such as comorbidities, detailed nutritional status, or systemic inflammatory markers were not systematically available. Temporal muscle measurements were performed according to a standardized protocol, yet inter-rater variability and differences in imaging acquisition across centers may affect reproducibility. We also used a median split to define low versus high muscle volume; while this approach facilitates comparison with previous studies, alternative cutoffs or sex-specific thresholds might yield different absolute risk estimates [18,19]. Finally, our analyses focused on overall survival; future research could explore the relationship between muscle volume, toxicity, quality of life, and functional outcomes.

Despite these limitations, our findings add to the accumulating evidence that preoperative temporal muscle metrics are clinically relevant biomarkers in glioblastoma. By demonstrating that bilateral temporal muscle volume is independently associated with overall survival after adjustment for age, functional status, surgery type, treatment, and molecular markers, we highlight the potential value of incorporating objective sarcopenia assessment into routine neuro-oncologic practice. Prospective multicenter studies are warranted to validate these results, define optimal measurement and cutoff strategies, and test whether interventions targeting sarcopenia and frailty can translate into improved outcomes for patients with glioblastoma.

## Conclusions

In this retrospective cohort of adults with glioblastoma, lower preoperative bilateral temporal muscle volume was consistently associated with shorter overall survival and remained an independent prognostic factor after adjustment for age, performance status, surgical approach, postoperative treatment, and molecular markers. Our findings support temporal muscle-based sarcopenia as a practical imaging marker of frailty that complements established prognostic variables and can be derived from routine preoperative cranial imaging without additional cost or patient burden. Incorporating temporal muscle volume into clinical assessment may help refine risk stratification, guide treatment intensity, and identify patients who could benefit from enhanced supportive care or prehabilitation. Prospective multicenter studies are warranted to validate these results, harmonize measurement and cutoff strategies, and evaluate whether interventions targeting sarcopenia and frailty can improve outcomes in glioblastoma.
